# Sympatric Apes in Sacred Forests: Shared Space and Habitat Use by Humans and Endangered Javan Gibbons (*Hylobates moloch*)

**DOI:** 10.1371/journal.pone.0146891

**Published:** 2016-01-20

**Authors:** Melissa Ann Reisland, Joanna E. Lambert

**Affiliations:** 1 Department of Anthropology, The University of Wisconsin, Madison, Madison, Wisconsin, United States of America; 2 Department of Anthropology, University of Colorado–Boulder, Boulder, Colorado, United States of America; University of Portsmouth, UNITED KINGDOM

## Abstract

In this research, we use a combination of ethnographic observation and GIS analysis to explore the use of space by humans and gibbons (*Hylobates moloch*) to determine areas of potential space competition in the sacred forest and nature reserve Cagar Alam Leuweung Sancang in West Java, Indonesia. More specifically, we test whether gibbons respond to the presence of humans in a manner consistent with predator-avoidance and predicted that the gibbon study subjects would avoid areas visited by humans (Risk-Disturbance Hypothesis). Data were collected August 2010-June 2011. We collected GPS locations and behavioral data on both the humans (6,652 hours) and the gibbons (1,253 hours) in the forest using 10 minute instantaneous sampling. Results indicate that humans preferentially assemble at the most sacred spot in the forest (Cikajayaan waterfall). Two gibbon groups’ home ranges encompassed most of the sacred areas. Group B avoided areas of high human use, as high human use areas and high gibbon use areas did not overlap. Group C, though, continued to use areas that were heavily visited by humans. We thus found partial support for the Risk-Disturbance Hypothesis, although the variation in gibbon response to human disturbance indicates behavioral flexibility. We suggest that understanding the effects of shared space on wildlife is necessary for informing conservation policy in human-visited forests.

## Introduction

A central goal of landscape ecology is to determine how spatial and temporal distribution of species influences community interactions, including competition for space [1]. While conflict between humans and other species over shared space has garnered much research attention, it is frequently from the perspective of animals entering human landscapes (e.g., crop raiding, predation), where costs to both humans and animals are evident [2–5]. Nature-based tourism is generally considered to be a low source of conflict between humans and wildlife, especially relative to crop raiding, hunting, or habitat destruction [6–9]. Nonetheless, human presence at tourist sites still influences animal behavior and the consequences of humans and animals sharing landscapes via nature-based tourism remain poorly understood for most species [6,7, 10–17].

Here, we employ Geographic Information System (GIS) to investigate the use of space by both humans and a highly-endangered sympatric ape species (Javan gibbons, *Hylobates moloch*) in a forest heavily used by spiritual tourists. Spiritual tourism is defined as tourism characterized by a self-conscious project of spiritual betterment [18]. Spiritual tourism to a natural area falls within the realm of nature-based tourism, in which wildlife or natural features draw tourists to a site, but where tourists may or may not contribute to long-term conservation [7]. Because space itself is the contested resource, GIS is a valuable means by which to evaluate the effects of human presence on gibbon behavior, thereby providing data useful for land planning and implementation of informed conservation tactics [19–21].

We explicitly evaluate the Risk-Disturbance Hypothesis (RDH). The RDH predicts that the short-term responses of animals to disturbance, such as human or predator presence, will be proportional to the perceived risk of the situation [12, 22]. Moreover, it predicts that animals will selectively use habitats that offer low risk to energy intake ratios and spend less time than expected in high-risk areas despite resource abundance. [12, 23–25]. Avoidance of perceived risky areas can have the same effect on a species as habitat loss or degradation because the animal underuses resources from the risky areas [26, 27]. For example, pink-footed geese (*Anser brachyrhynchus*) exploit feeding fields less (obtain less food per field) as the risk of human disturbance to the field increases [27] and elk (*Cervus elephus*) decrease their feeding time when closer to roads [28]. Population decline can result from reduced efficiency of exploiting food resources, reducing reproductive output [24, 29, 30].

In West Java, where Javan gibbons are endemic, there is a long history of humans using forest habitat for both resource extraction and cultural reasons [31], including spiritual visits to the many sacred forests [32]. The extremely high population density of this land, as well as the history of forestland conversion, has resulted in many populations of Javan gibbons living in highly fragmented habitats that frequently overlap with human land use [33, 34]. Though human disturbance (resulting from human interaction with natural areas) does not typically result in mortality, animals living in fragments are unable to alter ranging patterns to obtain resources elsewhere [13, 26].

We compare use of a sacred forest by Javan gibbons, villagers, and Indonesian spiritual tourists who entered the forest to determine whether human presence limits gibbons’ access to key food and spatial resources [26]. We test the hypothesis that if human presence affects gibbon ranging patterns, gibbons will alter their ranging patterns to avoid human disturbance. Animals commonly respond to human disturbance in two ways, by altering their habitat use patterns to avoid direct contact with humans (i.e. avoiding areas where humans are present) [35–40], or by avoiding or underusing areas of repeated human disturbance (i.e. avoiding areas of frequent human use, both when humans are absent and present) [12, 27, 41–43]. We evaluate two predictions that test each of these potential avoidance strategies in Javan gibbons. To our knowledge, there are no existing data on sympatric interactions among humans and gibbons, although it remains a crucial topic of conservation concern as range overlap and shared space continue to expand.

**Prediction 1:** If disturbance from human forest use affects gibbon ranging patterns, gibbons will avoid areas when human presence increases. Specifically, we tested whether the number of humans present at a location at a given time affects the likelihood of observing a gibbon at that time. We expected as the number of humans present increases in an area, the likelihood of gibbons also being present would decrease.

**Prediction 2:** If areas of heavy human disturbance are perceived as risky by gibbons, gibbons will underuse such areas relative to other home range areas. Based on our first prediction, gibbons may avoid areas when humans are present, but they may also avoid areas humans commonly use, even when humans are absent. We located areas of high human use by recording the number of people both in the presence and absence of gibbons to determine areas of high human use independent of gibbon presence. We expected the average number of humans present in an area would negatively correlate with the number of times a gibbon group was observed in the area, and forest use patterns for both gibbons and humans would differ, as gibbons would underuse areas of high human use.

## Materials and Methods

### Ethics Statement

This research complied with the protocols of the Institutional Animal Care and Use Committee of the Research Animal Resource Center (research exempt from protocol as a wildlife observational study), and the Institutional Review Board for human subjects research (protocol number SE-2008-0210) at the University of Wisconsin, Madison. Our data collection complied with federal laws of Indonesia. Appropriate permits were obtained to enter and conduct research at the nature reserve Cagar Alam Leuweung Sancang from the Indonesian Forestry Department (# SI 203), the Indonesian Department of Research and Technology, and the Indonesian Police Department (# 00-182620/p0/VII/2010).

### Study Site

**Fig 1:**
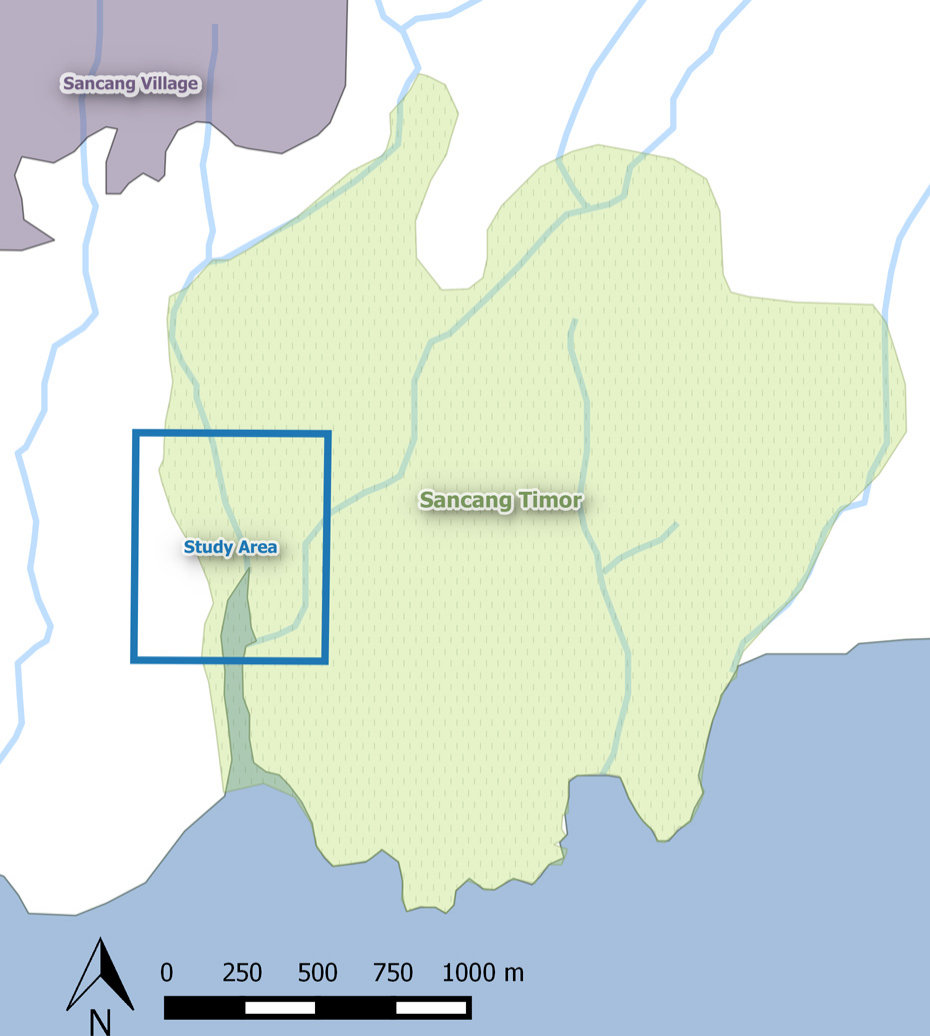
Sancang Timur and Study Area. Generated using OpenStreetMap data (modified to add rivers).

This study was part of a larger, long-term project on the behavioral ecology and conservation biology of the endangered Javan gibbon. All field research was conducted at the nature reserve and sacred forest Cagar Alam Leuweung Sancang (CALS), located in West Java, Indonesia. CALS is a 2,157 hectare reserve located in the province Garut on the south coast of West Java, established in 1978 by the Indonesian Forestry Department to protect lowland dipterocarp and mangrove forests and endangered and endemic flora and fauna. A heavily logged area divides the reserve into two fragments: Sancang Timur (East Sancang; 2km^2^) and Sancang Barat (West Sancang; 4km^2^) [44]. CALS is protected by the Indonesian government as a nature reserve, and thus a permit is required to enter the forest. However, illegal human activity occurs daily, mostly in the form of spiritual tourism, and walking paths are present throughout the forest. An average of 16.73 people per day enter the forested area of the reserve within the ranges of several gibbon groups [44]. All data were collected in the Sancang Timur (Fig 1). Due to human activity, the study area contains multiple structures and landscape elements heavily frequented by humans. We assigned names to these areas and recorded their position. Areas of interest include: (1) The *Cikajayaan* waterfall, the most sacred site in the forest and most commonly visited area in the forest; (2) *Camping Site 1*, a structure with sleeping shelters for spiritual tourists, west of the Cipangisikan river on the main path to the riverbank; (3) *River Ferrying* site, a site at the river where humans are ferried across on a raft; (4) *Camping Shelters 2*, another structure with sleeping shelters south of Cikajayaan; (5) *Group B River Crossing*, a site where one of the gibbon groups crosses the Cipangisikan river, and (6) the *Forest Entrance*, the location where the forested area and path to the sacred sites begins.

### Study Subjects

#### Gibbons

An estimated 21–27 individual Javan gibbons live in 8–9 groups within CALS [44, 45]. Six to seven gibbon groups live in Sancang Timur and two groups in Sancang Barat. Though we observed six groups in Sancang Timur (Groups A-F), data for this study are limited to two groups, groups B and C (Table 1 for group composition) as the home ranges of only these groups overlap with the sacred areas in the forest. Group B’s home range is 13.5 ha. [44] and encompasses the entrance to the forest, the main path through the forest, the sacred site containing Camping Shelters 1, and the River Crossing site. Group C’s home range is 15 ha. [44] and encompasses the Cikajayaan waterfall, the River Crossing site, and Camping Shelters 2. The home range of both groups is larger than what we observed in this study because we focused our data collection on areas that were easily accessible to and frequently visited by humans. Group B’s home range extends further north along the Cipangisikan River along both banks than observed and Group C’s home range extends further east into the reserve [44]. Because visitors and locals have been using this forest for generations, gibbons have frequent human contact, do not flee upon seeing humans and are considered passively partially habituated to human presence [44].

**Table 1:** Study Groups in Sancang Timur.

Group	Location	Gibbon	Age/Sex
**B**	Footpath to river,	Tono	Adult Male
	W. of river and E.	Tini	Adult Female
	riverbank	Udian (dis. 11/10)	Juvenile
**C**	Cikajayaan and above,	Jay	Adult Male
	E. of river and N. of	Ann	Adult Female
	Cikajayaan	Cika	Juvenile Male
		Wana (born 5/11)	Infant

Though the dense understory and hills and valleys within CALS make the terrain difficult for humans to traverse, it is ideal for the brachiating gibbons. All groups can move easily within the contiguous forested areas of their home ranges. The multiple human-made structures throughout the forest do not appear to greatly impede gibbon travel because the canopy remains connected. However, gibbon movement is restricted in some ways. Major geographic barriers such as the ocean to the south and the afore mentioned Cipangisikan River serve as home range borders, as do the areas cleared by logging and the home ranges of other gibbon groups.

#### Humans

The majority of people entering the reserve are Indonesian spiritual tourists, mostly from West Java, who come to the forest seeking a change in luck [45]. There is wide variation in the duration of a spiritual tourist visit (1 hour—several months), although many are one night or less (mean = 2.3 days, n = 73, SD = 1.7). Locals from the village of Sancang also enter the forest for non-spiritual reasons including resource extraction (fishing, hunting, removing timber), acting as porters and guides for spiritual tourists, or acting as *kuncen*, or spirit master, who mediate the interactions between the spiritual tourists and the spirit world [45]. Human activity varies by time of day, day of the week, and time of the year, but we observed either a spiritual tourist or local in the forest every observation day. The majority of people entering the forest are spiritual tourists, and villagers that enter the forest often do so accompanying spiritual tourist groups (acting as porters and guides). Thus, most encounters we observed between humans and gibbons occurred with spiritual tourists present. While villagers occasionally extracted resources from the forest, spiritual tourists rarely did. The density of humans within the forest therefore did not likely impact gibbon resource distribution. Both spiritual tourism and local activity in the forest is technically illegal because neither spiritual tourists nor Sancang locals obtain permits, but the local Forestry officials do not prevent people from entering.

### Data Collection

Gibbon behavioral data were collected data from August 2010 until June 2011 (271 total days) with the assistance of 4 Indonesian field assistants. Each day, one or two researchers collected location data. If two researchers were collecting data for the day, each data collector positioned him or herself in the home range of a different group (in some locations, more than one group’s home range could be observed). Typically, only one researcher was present during data collection periods, as others were spread out among the six groups living in the forest. Data collection began between 0545 and 0615, or as soon as the researcher reached the assigned home range, and continued until 1630, or until the gibbons moved into their sleeping trees and stopped their activity. Gibbons were located either following their long call vocalizations or by searching their home range. Though we always attempted to locate or follow the gibbon groups throughout the day, because of forest topography we often did not know the location of one or more of the study groups. However, we collected a data sample instantaneously every 10 minutes in both the presence and absence of gibbons. When gibbons were not present, we collected GPS coordinates, recorded which gibbon groups' home ranges were visible, and recorded the number of humans visible (minimum of one, the data collector). Thus, data on the number of humans present at a location was collected independently from gibbon observations. Because most human groups encountered were either all tourists, or a mix of tourists and Sancang villagers, we only considered total number of humans observed. When gibbons were present, along with the above information, we also recorded the group present.

Prey species will engage in risk avoidance behaviors once they are able to detect a perceived risk [22]. Upon visual detection, Javan gibbons are known to respond to humans similarly to how they respond to predators [45,46]. Therefore in this study we use visual detectability to delineate shared space between humans and gibbons. We used our ability to detect humans as a proxy for what the gibbons could detect. Thus, if gibbons and humans were both visible in the same sample, they were considered to be in a shared space.

GPS data were collected using a Pharos 565 PDA with a built-in GPS receiver and a Garmin e Trex® GPS unit. Data were recorded using the Cybertracker Data Collection program [47]. GPS accuracy was recorded by Cybertracker using Dilution of Precision (DOP). DOP scores over 10 are considered to have only fair accuracy and were discarded from the analysis [48]. Only one GPS value was recorded per scan since gibbons typically remain in cohesive groups. Thus, when gibbons were present the entire scan only had one associated GPS coordinate regardless of how many gibbons in the group were visible.

### Analysis

One challenge we encountered in this study was mitigating and understanding the effects of human researchers on gibbon behavior as at least one human was always present during data collection. However, because we encountered human groups of various sizes, we treated the number of humans present as a continuous variable and measured gibbon response to the differences in human group size. Though we cannot control for human presence completely, we tested our hypotheses against change in the intensity of human presence. Even animals regularly exposed to human presence may alter their behavior, especially in areas where the degree and intensity of human presence varies spatially [38, 49]. Thus, although we cannot know how the gibbons behave in the complete absence of human observers, we can still determine whether a relationship exists between human and gibbon spatial use patterns.

#### Prediction 1

We used logistic regression analysis to determine whether gibbons were less likely to be in a particular area as the number of people increased. For each group, we included all scans where that group’s home range was visible except for scans that included data from gibbons not in the focal group (from several locations multiple home ranges were visible). Scans where gibbons were present were coded as 1 and scans where gibbons were absent were coded as 0. We performed logistic regression of gibbon presence against the number of people present per scan. Due to limited samples, scans with over 15 people were excluded from the analysis. These analyses were performed with JMP statistical software version 9.0 (JMP™, Cary, NC).

#### Prediction 2

We imported GPS values into the ArcGIS 10 and 10.1 ArcMap software (ESRI, Redlands, CA). We analyzed data from groups B and C separately. To establish the known range of each group we used only the GPS points where gibbons were present and projected the points on to a Bing base map (Microsoft, 2012). All points outside the research area were manually deleted (e.g. points in water, points in cleared areas, points outside the study area). We merged the point data to a 15m x15m grid. We only displayed cells that contained points (i.e. cells where gibbons were observed at least once).

For each group, we imported the points (both gibbons present and absent) when scans were conducted in the visible area of that group's home range. As more than one home range was visible from many locations, we removed all data when gibbons other than the focal group were visible. We manually removed all data points that fell outside of the previously established known ranges (cells where gibbons were observed). We merged these points on to the 15m x15m grid already created. For number of humans, the value for each cell represented the mean number of humans seen in that cell. For gibbon data, the value of each cell represented the sum of the total number of times gibbons were seen in that cell. This created a map for the observed home range of both gibbon groups.

We used Ordinary Least Squares (OLS) regression to evaluate whether the average number of humans present in a cell was correlated with the number of times gibbons were observed in that cell. We created several OLS models using exploratory regression analysis with gibbons present as the dependent variable and different fixed explanatory variables including number of humans, number of researchers, number of spiritual tourists and locals, presence of spiritual tourists or locals in the scan, distance from forest edge, distance from river, distance from noted forest sites, and distance from human researchers. We eliminated all non-significant variables, and used Akaike’s Information Criterion (AIC) to determine which model had the best fit. AIC is a relative measure of model performance that allows for comparison of models with the same dependent variable. Lower AIC scores of at least three indicate a better fit model [50]. The best fit model used total humans only as the explanatory variable to predict gibbon presence, so we removed all other factors from the model.

However, spatial autocorrelation is a known problem with OLS regression models using geographic data, which invalidates the independence assumption of these models [51]. As spatial autocorrelation occurred in our data, we also ran a Geographically Weighted Regression (GWR) which accounts for spatial autocorrelation by evaluating each feature separately within the context of a particular bandwidth, or neighborhood, resulting in localized relationships between the predictor and explanatory variables [52]. We used an adaptive GWR model with AIC scores to determine the bandwidth for each feature. We used total humans as the only explanatory variable since it was the best fit OLS model.

We used the hotspot analysis tool to find statistically significant spatial clustering of higher or low values for humans and gibbons. These analyses find spatial clustering by comparing values of a fixed area or point to those in its surrounding neighborhood. In this case, each 15m x15m cell had a value for average number of humans per cell and a total number of scans with gibbons present per cell. Hotspot neighborhoods were created with a fixed distance band of 45m. The analyses output Z-scores, which measure clustering. High positive Z-scores indicate clustering of high values (red hot spot) and high negative Z-scores indicate a clustering of low values (blue cold spot). Z-scores near 0 indicate no significant clustering (beige) [52]. Each Z-score is associated with a P value and the output categories correspond to various P values. Table 2 relates Z-scores to their corresponding P values. For each group, we ran hotspot analysis using values for mean number of humans per cell, and the total number of scans with gibbons present per cell.

**Table 2:** Z-scores and corresponding p-values for Hotspot analysis in ArcGIS.

Z-score	P-value
<-2.58	0.01
-2.58–1.96	0.05
-1.96–1.65	0.10
-1.65–1.65	NS
1.65–1.98	0.10
1.98–2.58	0.05
>2.58	0.01

To find areas where human use and gibbon use of the forest differed, we converted the Z-score polygons of the hotspot analyses into rasters and subtracted the gibbon Z-scores for each cell from the corresponding human Z-scores from the same cell. This resulted in cells with high human use but low gibbon use having a high positive (red) value and cells with high gibbon use and low human use having a high negative value (blue). Cells where human use and gibbon use of an area were similar (either high or low) had a value close to zero.

## Results

The total area in which Group B was observed was 6.59 ha. The total area in which Group C was observed was 2.32 ha. This size represents the total area of all cells in which gibbons were observed, but not the total home range nor the area in between cells where gibbons were not observed, although these regions are likely part of the gibbons’ true home range. Here, we address each of the predictions as outlined in the introduction.

### Prediction 1: If disturbance from human forest use affects gibbon ranging patterns, gibbons will avoid areas when human presence increases

In this section, we analyzed whether gibbons were less likely to be seen in an area as the number of people there increased. As the number of people observed per scan increased within Group B’s home range, members of Group B were less likely to be present for the scan (n = 23733, β = 0.2372, S.E. = 0.015, P<0.0001; positive β indicates decreasing likelihood of gibbon presence). As the number of people observed per scan in Group C’s home range increased, Group C gibbons were not more or less likely to be present (n = 18788, β = -0.014, S.E. = 0.011, P = 0.21).

### Prediction 2: If areas of heavy human disturbance are perceived as risky by gibbons, gibbons will underuse such areas relative to other home range areas

**Fig 2:**
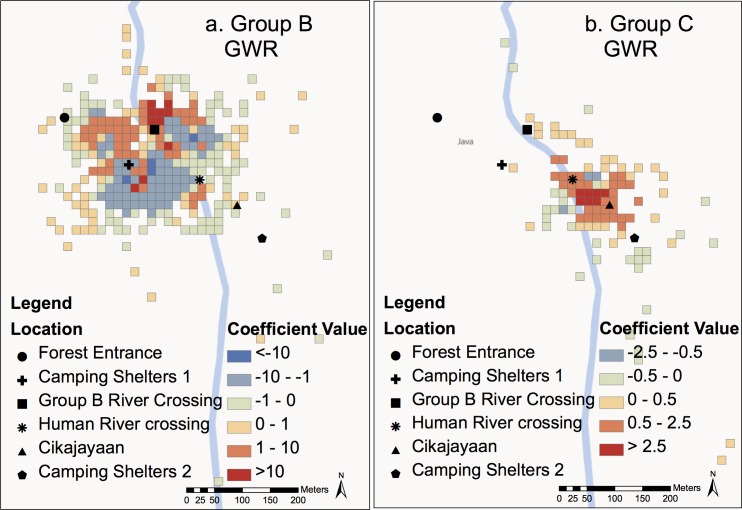
Global Weighted Regression (GLR) analysis for groups B (a) and C (b). Total number of gibbon observations per cell is the dependent variable and average number of humans per cell is the explanatory variable. Red cells indicate a positive relationship (coefficient value) between the variables and blue cells indicate a negative relationship (coefficient value)

In this section, we compared human forest use with gibbon forest use. OLS regression analysis indicates a negative correlation between the average number of humans observed per cell and the number of times gibbons were observed for Group B (Table 3). However, the overall fit of this model is low due to spatial autocorrelation. The GWR model for Group B (Fig 2A) shows that in most areas there is a weak to moderate negative relationship between number of humans and number of gibbon observations, but at some areas, specifically near Group B River Crossing and Camping Shelters 1, there is actually a positive relationship between number of humans and number of gibbon observations.

**Table 3:** Ordinary Least Squares (OLS) and Geographically Weighted Regression (GWR) scores for Groups B and C with Total number of times gibbons were observed per cell as the dependent variable and average number of humans per cell as the explanatory variable.

	Group B		Group C	
	OLS	GWR	OSL	GWR
**Coefficient**	-0.882267	Fig 2	0.65134	Fig 2
**R**^**2**^ ^**a**^	0.030117	0.70587	0.056243	0.2718
**AIC**^**b**^	1711.71	1601.42	534.078	526.57
**Wald Statistic**^**c**^	32.2489	NA	7.705184	NA
**P Value**	<0.00001	NA	0.005506	NA

^a^ Represents adjusted value

^b^ Akaike’s Information Criterion. A relative score that measures the fit of the model. Within each group, a lower score of at least 3 indicates a better fit model

^c^ Non normal distribution

Both OLS regression and GWR for Group C indicate a moderate positive relationship between the average number of humans per cell and the total number of gibbons observed, however the GWR model does provide a slightly better fit (Table 3; Fig 2B). Only a few areas on the periphery of the observed range show a slight negative relationship between number of humans and number of gibbon observations. The number of humans per cell explained more of the variation in number of gibbon observations for Group B than for Group C.

**Fig 3:**
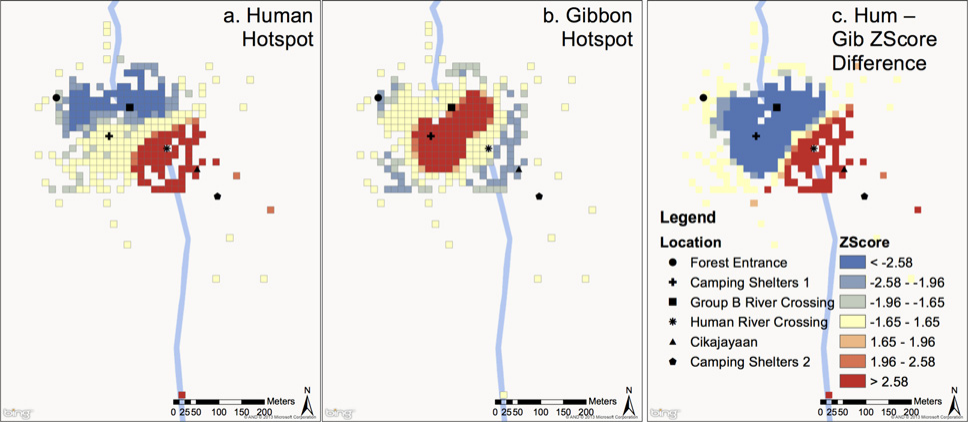
Hotspots for humans (a) and group B gibbons (b) in group B’s home range. Colors correspond to Z-Scores in legend. Z-scores correspond to p-values from Table 1. Red color indicates clustering of high values (commonly used spot) and blue color indicates clustering of low values (infrequently used spot). For difference in human and gibbon Z-Scores (c) (human scores minus gibbon scores), blue areas indicate areas with high gibbon scores and low human scores (favored by gibbons but avoided by humans) and red areas indicate areas of high human scores and low gibbon scores (favored by humans and avoided by gibbons) Beige areas are areas where Z-scores for humans and gibbons are similar (either low or high). Cells are 15mx15m.

Fig 3 shows hotspots within Group B’s home range for both humans (a) and Group B gibbons (b). The home range of Group B is on the west side of the Cipangisikan River, but crosses to the east side of the river north of Cikajayaan. All points on the east side of the river at or south of Cikajayaan were taken while viewing gibbons at the riverbank from across the river. Humans cluster near the riverbank at and just south of the *River Ferrying* site and around *Cikajayaan*. Gibbons cluster at *Camping Shelters 1* west of the river and at *Group B River Crossing*, north of Cikajayaan (Fig 3). Fig 3 also shows the difference in Z-scores between human clustering and gibbon clustering (human scores minus gibbon scores) for groups B (c). The large red and blue areas in Group B’s home range indicate that gibbons and humans use largely different parts of the home range, with gibbons concentrated in the northwest portion near the *Camping Shelters 1* and *Group B River Crossing* site, and humans in the southeast near the *River Ferrying* site and *Cikajayaan*. There is a small overlap zone between the *Group B River Crossing* site and the *River Ferrying* site that is a high use area for both (beige area).

**Fig 4:**
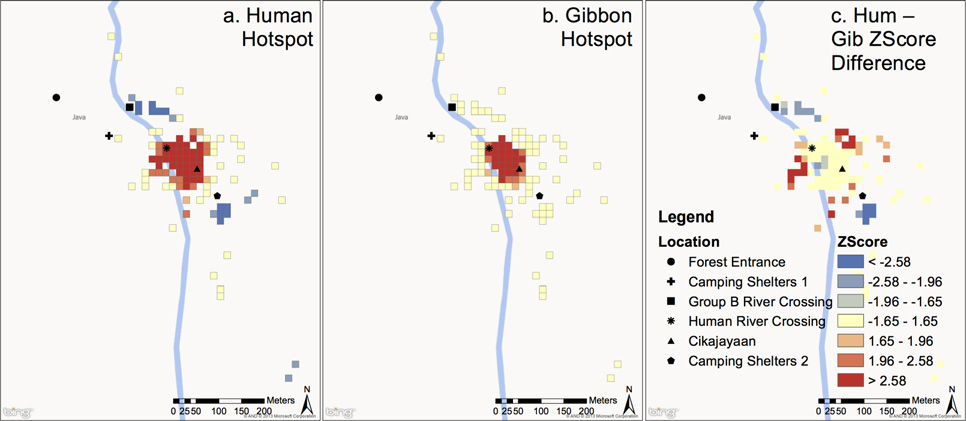
Hotspots for humans (a) and group C gibbons (b) in group C’s home range. Colors correspond to Z-Scores in legend. Z-scores correspond to p-values from Table 1. Red color indicates clustering of high values (commonly used spot) and blue color indicates clustering of low values (infrequently used spot). For difference in human and gibbon Z-Scores (c) (human scores minus gibbon scores), blue areas indicate areas with high gibbon scores and low human scores (favored by gibbons but avoided by humans) and red areas indicate areas of high human scores and low gibbon scores (favored by humans and avoided by gibbons) Beige areas are areas where Z-scores for humans and gibbons are similar (either low or high). Cells are 15mx15m.

Fig 4 shows hotspots within Group C’s home range for both humans (a) and gibbons (b), Group C’s home range is strictly on the east side of the Cipangisikan river. Any observations from the west side of the river were taken while viewing gibbons on the riverbank from across the river. Group C’s home range extends eastward, but lack of accessibility restricted observations in this area. Both humans and gibbons clustered around *Cikajayaan* and the *River Ferrying* site (Fig 4). Fig 4 also shows the difference in Z-scores between human clustering and gibbon clustering (human scores minus gibbon scores) for group C (c). There are no obvious differences between where gibbons use the forest and where humans use the forest, indicated by the large beige colored areas. However, high gibbon use and low human use occurred at the extreme northern and southern most parts of the range, whereas high human use and low gibbon use occurred at the extreme eastern and western most parts of the range.

## Discussion

Our first prediction suggested that Javan gibbons would be less likely to be observed in an area as the number of people in that area increased. This prediction was partially met. Sighting frequency (how often animals are observed in areas of varying disturbance) is commonly used to measure the impact of human disturbance on animal populations [49,53,54] and the method used in this study allowed us to see the affects of human group size on gibbon presence. Group B and Group C responded differently to human presence in their home range. Group B supported our hypothesis and was much more sensitive to human presence. They were less likely to be present in a scan when more people were visible. Group C, however, did not meet the prediction, and was just as likely to be observed with increasing numbers of people present.

However, these data alone do not provide information about gibbon and human preferred use of space. Some animal species are known to completely avoid human-visited areas, even when humans are absent [53, 55, 56] and even when the animals are otherwise habituated [49]. Therefore, our second prediction suggested that if areas of high human use are perceived as risky by gibbons, gibbons will avoid such areas at all times. Again, this was partially supported. Group B and Group C used their home ranges differently with respect to human use. For group B, the number of scans per cell with gibbons present was negatively correlated to the average number of humans per cell, indicating they underuse areas with greater human presence. The GWR analysis did display areas where the number of humans positively affected the number of gibbon observations, but these areas largely occurred in areas where the average number of humans per cell was low (human cold spots), and therefore had little variation in the total number of humans present. Thus, the positive correlation likely results from more gibbon present scans with only slight increases in human presence (e.g., gibbons present in situations where there were only two humans instead of one). Within Group B’s home range, humans and gibbons preferentially used different parts of the forest, supporting our prediction. Human use clustered at the *River Ferrying* site, and gibbon use clustered at the *Camping Shelters 1* site and the *Group B River Crossing* site with little overlap between preferred gibbon areas and preferred human areas. As human forest use patterns are dictated by the location of the sacred sites, these results, coupled with the avoidance and regression results, suggest that Group B used the forest in a non-random way to avoid humans.

However, for group C, both OLS regression and GWR show a slight positive relationship between the number of humans per cell and the number of scans with gibbons present, though the lower coefficient values and r^2^ values indicate that number of humans was not as robust of a predictor for gibbon presence as it was for Group B. Group C gibbons and humans both preferred to use the part of their home range concentrated around the *Cikajayaan* waterfall; these observations do not support our hypothesis. We observed a large degree of overlap between preferred gibbon space use and preferred human space use. This, again coupled with the avoidance and regression data, suggests that Group C gibbons did not alter their forest space use with respect to human space use.

One important caveat must be addressed with the data from Group C. While we had access to nearly all of Group B’s home range, much of Group C’s home range was inaccessible to us and thus the majority of areas where Group C could be visible were also areas of high human concentration (located near *Cikajayaan* waterfall). This led to a bias in sampling in which more accessible areas were oversampled relative to inaccessible areas. Despite this, there were often times when the area around *Cikajayaan* was empty of humans. Group C could have easily avoided the area when crowded and returned when groups of people left, but they used the area equally or more as the number of people increased.

While it is possible that these results are impacted by the limited accessibility of Group C’s home range, as addressed above, it is important to note that only areas where gibbons were observed were recorded on the maps. We performed scans in many other parts of Group C’s presumed home range where human presence was less intense, and never observed Group C. Additionally, although data from Group C only reflects the accessible part of their home range, their use of the area is not uniform, indicting preferred areas of use within the visible range. Thus, while we cannot account for all of Group C’s home range use, our data do reflect a true overlap in preferred space between gibbons and humans.

It is also possible that differences in group composition may account for some observed differences between the groups. Group B contained an independent juvenile for four months of the study period (July–October, when the juvenile disappeared), and an infant was born into group C at the end of the study (observed May–June). However, both groups consisted of an adult male/female pair for the majority of the study, and both groups were also observed with an immature gibbon. Therefore, we do not suspect group composition can account for the differences observed between the two groups; moreover, inter-group comparisons would be challenged by the few data collected when both groups comprised three individuals. Group composition cannot be ruled out as an explanatory variable, though, and further research is necessary to determine the role it may play in spatial distribution.

In addition to being consistent with the RDH, data presented here contribute to an emerging picture of gibbon behavior suggesting more behavioral flexibility [44, 57] than has been described previously [58]. The two study groups responded differently to the presence of humans, with Group C having a much weaker reaction to the humans in their home range. Although disturbance is higher at Cikajayaan, Group C gibbons may perceive the risk as lower because of increased habituation, or the trade-off of avoiding the area may be too great because of the location of important feeding trees. Recent work on gibbon behavior has demonstrated that gibbons have the ability to vary their diet and social structure [57] and exploit edge habitat [44]. In addition, this study indicates that gibbons are able to adjust their behaviors to various levels of human disturbance.

Despite potential range restrictions and pressures from human use, the current density of gibbons in the Sancang Timur fragment of CALS is consistent with other sites at 3.00 groups/km^2^ (2.5–3.8 groups/km^2^ [46, 59–62]. However, a lag time in response to anthropogenic stress cannot be ruled out. Spiritual tourists have been visiting the Sancang forest for generations [32] (Sancang villagers, pers. comm), but it is only since the early 2000s that the fragments Sancang Timur and Sancang Barat have been separated, restricting the gibbons’ ability to travel from one to the other [44]. Habitat loss and fragmentation has resulted in space becoming a limiting resource for many species [63]. A common animal response to human disturbance is to leave the area of disturbance [26, 38], but the gibbons in Sancang Timur cannot leave, even to Sancang Barat, because of recent habitat fragmentation. Thus, the long-term consequences of human disturbance on this population of gibbons are not yet fully understood. Continuous human presence, even in the form of long-term research only, potentially results in shifts in primate behavior and substrate use [64–66]. The consequences for Javan gibbons may be reflected in the fact that the fragment has shown no population growth since censusing after the fragmentation began in 2004–2005 [67]. Additionally, this study did not specifically test resource abundance. Though the spiritual tourism in the forest seemingly does not impact resource abundance, the presence of large human groups may greater effect gibbon ranging patterns if the location of areas of high human density correspond to areas of high resource abundance. This is likely the case with group C, which regularly used the area around the Cikajayaan waterfall, despite high human density.

In this research, we demonstrate the effectiveness of using GIS analysis as a tool for evaluating shared space between animals and humans and integrating human presence into landscape ecology models. Using a GIS approach, we were able to examine and compare human and gibbon use simultaneously over the entire landscape as well as to parse individual behaviors and localized distribution patterns. Moreover, we were able to test the risk-disturbance hypothesis by getting a clear picture about areas of overlap between humans and gibbons as well as areas of avoidance by one or the other. Recognizing that human/animal shared space will remain the reality under which conservation policy will be made [68], this type of analysis allows us to chart and manage specific areas within a larger landscape and determine sustainable ways for humans and other species to share habitats.

## Supporting Information

S1 Dataset Contains data collected for this study. Only data in the tab labeled scan with accuracy BC were used in the analyses.
(XLSX)
